# Supplementary material for: The Life cycle Assessment Integrated with the Lexicographic Method for the Multi-Objective Optimization of Community-Based Rainwater Utilization
Source: Int J Environ Res Public Health. 2023 Jan 25;20(3):2183. doi: 10.3390/ijerph20032183 (PMC9916029; doi:10.3390/ijerph20032183)
Supplement: Supplementary file 1 [file ijerph-20-02183-s001.zip › ijerph-2116116-supplementary.pdf]

International Journal of
Environmental Research and Public Health

MDPI

Supplementary Materials

The Life Cycle Assessment Integrated with the Lex-Icographic Method for the Multi-Objective Opti-Mization of Community-Based Rainwater Utiliza-Tion

Yi Li[1], Wenjun Xu[1], Wenlong Zhang[1,*], Youyi Huang[2], Fenfen Wan[1] and Wei Xiong[3]

[1] Key Laboratory of Integrated Regulation and Resource Development on Shallow Lakes, Ministry of Education, College of Environment, Hohai University, Nanjing 210098, China
[2] School of Architecture and Civil Engineering, Xiamen University, Xiamen 361000, China
[3] School of Hydraulic and Environmental Engineering, Changsha University of Science & Technology, Changsha 410114, China
* Correspondence: 1223zhangwenlong@163.com

Supplementary Figures

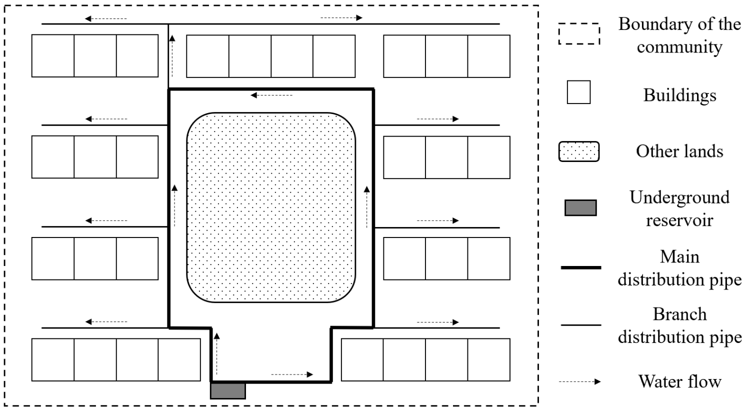

(a)

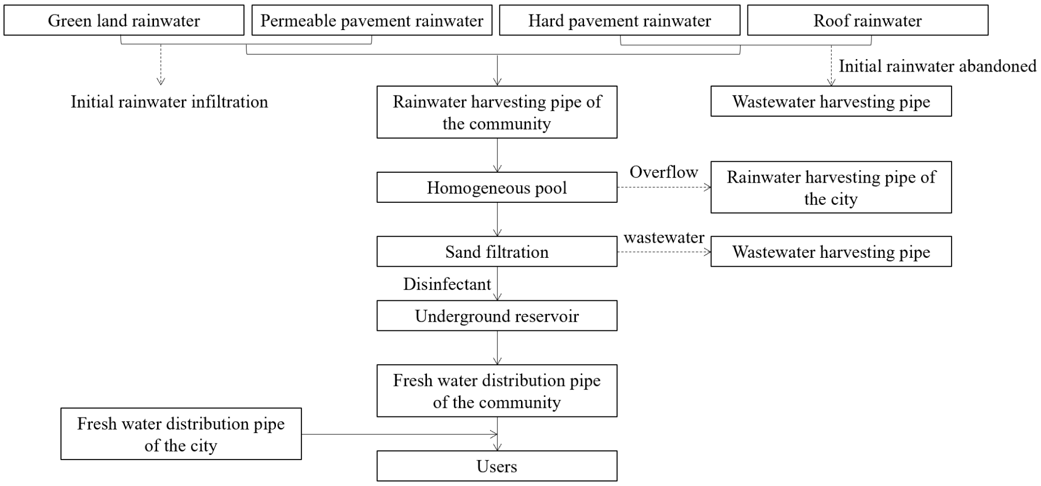

(b)

Figure S1. The rainwater harvesting and utilization system of a community. (**a**) schematic of residential buildings layout and pipeline network, (**b**) flow chart for community-based rainwater harvesting and utilization pattern.

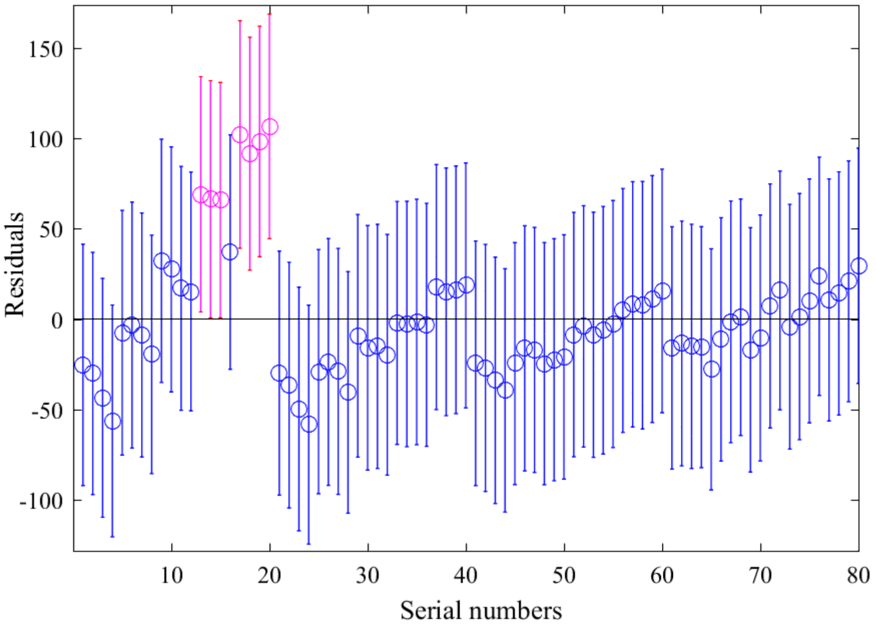

Figure S2. Residual plot of fitting for abiotic depletion potential for fossil fuels formula.

Supplementary Tables

Table S1. Engineering scale for all scenarios of community-based rainwater harvesting and utilization.

Floor-Area Ratio (FAR)	1									
**Total Runoff Coefficient**	**0.09**					**0.20**				
**Reservoir Volume (m³)**	**33**	**66**	**99**	**132**	**165**	**33**	**66**	**99**	**132**	**165**
**Storage system**										
1. Homogeneous pool (Length × Breadth × Height, m)	4 × 4 × 2.1	5 × 6 × 2.2	6.6 × 5 × 3	8 × 5.5 × 3	9.2 × 6 × 3	4 × 4 × 2.1	5 × 6 × 2.2	6.6 × 5 × 3	8 × 5.5 × 3	9.2 × 6 × 3
**Treatment system**										
2. Filtration pool (L × B × H)	1 × 0.85 × 1 m									
3. Underground reservoir (L × B × H)	9.2 × 6 × 3 m									
4. Disinfectant (kg)	68	73	83	88	92	94	103	122	137	148
**Distribution system**										
5. Main distribution pipe	Steel pipe, DN200, 370 m									
6. Branch distribution pipe	Steel pipe, DN100, 520 m									
7. Main household distribution pipe	PPR pipe, DN90, 360 m									
8. Branch household distribution pipe	PPR pipe, DN25, 6480 m									
9. Water pump	IS65-40-200 (2900), 56 kg in weight									

Table S1. Engineering scale for all scenarios of community-based rainwater harvesting and utilization (continued).

**Floor-Area Ratio (FAR)**					**1**					
**Total Runoff Coefficient**			**0.31**					**0.42**		
**Reservoir Colume (m³)**	**33**	**66**	**99**	**132**	**165**	**33**	**66**	**99**	**132**	**165**
**Storage system**										
1. Homogeneous pool (L × B × H, m)	4 × 4 × 2.1	5 × 6 × 2.2	6.6 × 5 × 3	8 × 5.5 × 3	9.2 × 6 × 3	4 × 4 × 2.1	5 × 6 × 2.2	6.6 × 5 × 3	8 × 5.5 × 3	9.2 × 6 × 3
**Treatment system**										
2. Filtration pool (L × B × H)					1 × 0.85 × 1 m					
3. Underground reservoir (L × B × H)					9.2 × 6 × 3 m					
4. Disinfectant (kg)	108	118	142	160	176	117	128	154	175	193
**Distribution system**										
5. Main distribution pipe					Steel pipe, DN200, 370 m					
6. Branch distribution pipe					Steel pipe, DN100, 520 m					
7. Main household distribution pipe					PPR pipe, DN90, 360 m					
8. Branch household distribution pipe					PPR pipe, DN25, 6480 m					
9. Water pump					IS65-40-200 (2900), 56 kg in weight					

Table S1. Engineering scale for all scenarios of community-based rainwater harvesting and utilization (continued).

**Floor-area ratio (FAR)**					**1.7**					
**Total runoff coefficient**			**0.09**					**0.20**		
**Reservoir volume (m³)**	**55**	**110**	**165**	**220**	**275**	**55**	**110**	**165**	**220**	**275**
**Storage system**										
1. Homogeneous pool (L × B × H, m)	5 × 5 × 2.2	7.4 × 5 × 3	9.2 × 6 × 3	14.7 × 5 × 3	15.3 × 6 × 3	5 × 5 × 2.2	7.4 × 5 × 3	9.2 × 6 × 3	14.7 × 5 × 3	15.3 × 6 × 3
**Treatment system**										
2. Filtration pool (L × B × H)					1.2 × 1.2 × 1 m					
3. Underground reservoir (L × B × H)					15.3 × 6 × 3 m					
4. Disinfectant (kg)	85	89	96	99	102	131	141	163	178	189
**Distribution system**										
5. Main distribution pipe					Steel pipe, DN200, 370 m					
6. Branch distribution pipe					Steel pipe, DN100, 520 m					

Floor-area ratio (FAR)	1.7									
Total runoff coefficient	0.09					0.20				
Reservoir volume (m³)	55	110	165	220	275	55	110	165	220	275
7. Main household distribution pipe	PPR pipe, DN90, 612 m									
8. Branch household distribution pipe	PPR pipe, DN25, 11016 m									
9. Water pump	IS65-40-200 (2900), 56 kg in weight									

Table S1. Engineering scale for all scenarios of community-based rainwater harvesting and utilization (continued).

Floor-area ratio (FAR)	1.7									
Total runoff coefficient	0.31					0.42				
Reservoir volume (m³)	55	110	165	220	275	55	110	165	220	275
**Storage system**										
1. Homogeneous pool (L × B × H, m)	5 × 5 × 2.2	7.4 × 5 × 3	9.2 × 6 × 3	14.7 × 5 × 3	15.3 × 6 × 3	5 × 5 × 2.2	7.4 × 5 × 3	9.2 × 6 × 3	14.7 × 5 × 3	15.3 × 6 × 3
**Treatment system**										
2. Filtration pool (L × B × H)	1.2 × 1.2 × 1 m									
3. Underground reservoir (L × B × H)	15.3 × 6 × 3 m									
4. Disinfectant (kg)	155	168	199	222	240	172	186	223	251	273
**Distribution system**										
5. Main distribution pipe	Steel pipe, DN200, 370 m									
6. Branch distribution pipe	Steel pipe, DN100, 520 m									
7. Main household distribution pipe	PPR pipe, DN90, 612 m									
8. Branch household distribution pipe	PPR pipe, DN25, 11016 m									
9. Water pump	IS65-40-200 (2900), 56 kg in weight									

Table S1. Engineering scale for all scenarios of community-based rainwater harvesting and utilization (continued).

Floor-area ratio (FAR)	2.4									
Total runoff coefficient	0.09					0.20				
Reservoir volume (m³)	78	156	234	312	390	78	156	234	312	390
**Storage system**										
1. Homogeneous pool (L × B × H, m)	5 × 6 × 2.6	8.7 × 6 × 3	13 × 6 × 3	20.8 × 5 × 3	21.7 × 6 × 3	5 × 6 × 2.6	8.7 × 6 × 3	13 × 6 × 3	20.8 × 5 × 3	21.7 × 6 × 3
**Treatment system**										
2. Filtration pool (L × B × H)	1.5 × 1.4 × 1 m									

**Floor-area ratio (FAR)**					**2.4**					
**Total runoff coefficient**			**0.09**					**0.20**		
**Reservoir volume (m³)**	**78**	**156**	**234**	**312**	**390**	**78**	**156**	**234**	**312**	**390**
3. Underground reservoir (L × B × H)					21.7 × 6 × 3 m					
4. Disinfectant (kg)	93	96	101	104	105	157	168	188	200	208
**Distribution system**										
5. Main distribution pipe					Steel pipe, DN200, 370 m					
6. Branch distribution pipe					Steel pipe, DN100, 520 m					
7. Main household distribution pipe					PPR pipe, DN90, 864 m					
8. Branch household distribution pipe					PPR pipe, DN25, 15552 m					
9. Water pump					IS65-40-200 (2900), 56 kg in weight					

Table S1. Engineering scale for all scenarios of community-based rainwater harvesting and utilization (continued).

**Floor-area ratio (FAR)**					**2.4**					
**Total runoff coefficient**			**0.31**					**0.42**		
**Reservoir volume (m³)**	**78**	**156**	**234**	**312**	**390**	**78**	**156**	**234**	**312**	**390**
**Storage system**										
1. Homogeneous pool (L × B × H, m)	5 × 6 × 2.6	8.7 × 6 × 3	13 × 6 × 3	20.8 × 5 × 3	21.7 × 6 × 3	5 × 6 × 2.6	8.7 × 6 × 3	13 × 6 × 3	20.8 × 5 × 3	21.7 × 6 × 3
**Treatment system**										
2. Filtration pool (L × B × H)					1.5 × 1.4 × 1 m					
3. Underground reservoir (L × B × H)					21.7 × 6 × 3 m					
4. Disinfectant (kg)	193	208	242	265	282	216	234	277	308	332
**Distribution system**										
5. Main distribution pipe					Steel pipe, DN200, 370 m					
6. Branch distribution pipe					Steel pipe, DN100, 520 m					
7. Main household distribution pipe					PPR pipe, DN90, 864 m					
8. Branch household distribution pipe					PPR pipe, DN25, 15552 m					
9. Water pump					IS65-40-200 (2900), 56 kg in weight					

Table S1. Engineering scale for all scenarios of community-based rainwater harvesting and utilization (continued).

**Floor-area ratio (FAR)**			**3.1**							
**Total runoff coefficient**			**0.09**					**0.20**		
**Reservoir volume (m³)**	**100**	**200**	**300**	**400**	**500**	**100**	**200**	**300**	**400**	**500**
**Storage system**										
1. Homogeneous pool (L × B × H, m)	8 × 5 × 2.5	13.4 × 5 × 3	20 × 5 × 3	22.3 × 6 × 3	21 × 6 × 4	8 × 5 × 2.5	13.4 × 5 × 3	20 × 5 × 3	22.3 × 6 × 3	21 × 6 × 4
**Treatment system**										
2. Filtration pool (L × B × H)	1.9 × 1.4 × 1 m									
3. Underground reservoir (L × B × H)	21 × 6 × 4 m									
4. Disinfectant (kg)	98	100	104	106	107	175	185	203	213	219
**Distribution system**										
5. Main distribution pipe	Steel pipe, DN200, 370 m									
6. Branch distribution pipe	Steel pipe, DN100, 520 m									
7. Main household distribution pipe	PPR pipe, DN90, 1116 m									
8. Branch household distribution pipe	PPR pipe, DN25, 20088 m									
9. Water pump	IS65-40-200 (2900), 56 kg in weight									

Table S1. Engineering scale for all scenarios of community-based rainwater harvesting and utilization (continued).

**Floor-area ratio (FAR)**			**3.1**							
**Total runoff coefficient**			**0.31**					**0.42**		
**Reservoir volume (m³)**	**100**	**200**	**300**	**400**	**500**	**100**	**200**	**300**	**400**	**500**
**Storage system**										
1. Homogeneous pool (L × B × H, m)	8 × 5 × 2.5	13.4 × 5 × 3	20 × 5 × 3	22.3 × 6 × 3	21 × 6 × 4	8 × 5 × 2.5	13.4 × 5 × 3	20 × 5 × 3	22.3 × 6 × 3	21 × 6 × 4
**Treatment system**										
2. Filtration pool (L × B × H)	1.9 × 1.4 × 1 m									
3. Underground reservoir (L × B × H)	21 × 6 × 4 m									
4. Disinfectant (kg)	222	238	271	293	307	254	273	319	351	374
**Distribution system**										
5. Main distribution pipe	Steel pipe, DN200, 370 m									
6. Branch distribution pipe	Steel pipe, DN100, 520 m									
7. Main household distribution pipe	PPR pipe, DN90, 1116 m									
8. Branch household distribution pipe	PPR pipe, DN25, 20088 m									

Floor-area ratio (FAR)					3.1					
Total runoff coefficient			0.31					0.42		
Reservoir volume (m³)	100	200	300	400	500	100	200	300	400	500
9. Water pump	IS65-40-200 (2900), 56 kg in weight									

Table S2. Life cycle assessment list of all scenarios for community-based rainwater harvesting and utilization.

Floor-area ratio (FAR)					1					
Total runoff coefficient			0.09					0.20		
Reservoir volume (m³)	33	66	99	132	165	33	66	99	132	165
**Construction phase**										
1. Polypropylene (PP, kg)	1.17E-01	1.37E-01	1.39E-01	1.46E-01	1.55E-01	9.09E-02	9.71E-02	9.34E-02	9.40E-02	9.64E-02
2. Quartz sand (kg)	1.39E-02	1.39E-02	1.24E-02	1.16E-02	1.11E-02	1.08E-02	9.89E-03	8.33E-03	7.46E-03	6.88E-03
3. Polyethylene (PE, kg)	6.61E-04	6.61E-04	5.87E-04	5.49E-04	5.26E-04	5.14E-04	4.70E-04	3.96E-04	3.54E-04	3.27E-04
4. Steel (kg)	7.91E-02	7.91E-02	7.02E-02	6.57E-02	6.30E-02	6.14E-02	5.62E-02	4.73E-02	4.24E-02	3.91E-02
5. Pentatricopeptide repeats (PPR, kg)	1.94E-02	1.94E-02	1.72E-02	1.61E-02	1.54E-02	1.51E-02	1.38E-02	1.16E-02	1.04E-02	9.58E-03
6. Polyvinyl chloride (PVC, kg)	3.82E-04	3.82E-04	3.39E-04	3.17E-04	3.04E-04	2.97E-04	2.72E-04	2.29E-04	2.05E-04	1.89E-04
7. Concrete (kg)	1.18E-01	1.41E-01	1.29E-01	1.36E-01	1.45E-01	9.16E-02	1.00E-01	8.73E-02	8.78E-02	9.00E-02
8. Geomembrane (kg)	1.36E-03	1.57E-03	1.51E-03	1.55E-03	1.61E-03	1.06E-03	1.12E-03	1.02E-03	1.00E-03	1.00E-03
9. Limestone (kg)	3.32E-01	3.32E-01	2.95E-01	2.76E-01	2.65E-01	2.58E-01	2.36E-01	1.99E-01	1.78E-01	1.64E-01

10. Water (kg)	4.61E+00	4.61E+00	4.10E+00	3.83E+00	3.68E+00	3.58E+00	3.28E+00	2.76E+00	2.47E+00	2.28E+00
**Operation and maintenance phase**										
11. Rainwater (kg)	1.00E+03	1.00E+03	1.00E+03	1.00E+03	1.00E+03	1.00E+03	1.00E+03	1.00E+03	1.00E+03	1.00E+03
12. Chlorine (kg)	1.00E-03	1.00E-03	1.00E-03	1.00E-03	1.00E-03	1.00E-03	1.00E-03	1.00E-03	1.00E-03	1.00E-03
**End disposal phase**										
13. Coal (kg)	5.74E-01	6.55E-01	6.53E-01	6.77E-01	7.14E-01	4.46E-01	4.65E-01	4.40E-01	4.37E-01	4.43E-01
14. Oil (kg)	8.41E-02	9.59E-02	9.56E-02	9.92E-02	1.05E-01	6.53E-02	6.82E-02	6.45E-02	6.40E-02	6.49E-02
15. Natural gas (m^3)	1.12E-02	1.28E-02	1.28E-02	1.32E-02	1.40E-02	8.71E-03	9.10E-03	8.61E-03	8.55E-03	8.67E-03
16. Electricity (kw·h)	4.57E-01	5.22E-01	5.20E-01	5.40E-01	5.69E-01	3.55E-01	3.71E-01	3.51E-01	3.48E-01	3.53E-01

Table S2. Life cycle assessment list of all scenarios for community-based rainwater harvesting and utilization (continued).

**Floor-area ratio (FAR)**	**1**									
**Total runoff coefficient**	**0.31**					**0.42**				
**Reservoir volume (m^3)**	**33**	**66**	**99**	**132**	**165**	**33**	**66**	**99**	**132**	**165**
**Construction phase**										
1. PP (kg)	7.95E-02	8.47E-02	8.05E-02	8.02E-02	8.13E-02	7.34E-02	7.81E-02	7.40E-02	7.34E-02	7.42E-02
2. Quartz sand (kg)	9.45E-03	8.63E-03	7.18E-03	6.36E-03	5.80E-03	8.73E-03	7.97E-03	6.60E-03	5.82E-03	5.29E-03
3. PE (kg)	4.49E-04	4.10E-04	3.41E-04	3.02E-04	2.76E-04	4.15E-04	3.78E-04	3.14E-04	2.76E-04	2.51E-04
4. Steel (kg)	5.37E-02	4.90E-02	4.08E-02	3.61E-02	3.30E-02	4.96E-02	4.53E-02	3.75E-02	3.31E-02	3.01E-02
5. PPR (kg)	1.32E-02	1.20E-02	1.00E-02	8.86E-03	8.08E-03	1.22E-02	1.11E-02	9.20E-03	8.11E-03	7.37E-03
6. PVC (kg)	2.59E-04	2.37E-04	1.97E-04	1.75E-04	1.59E-04	2.40E-04	2.19E-04	1.81E-04	1.60E-04	1.45E-04

7. Concrete (kg)	8.01E-02	8.74E-02	7.53E-02	7.48E-02	7.59E-02	7.40E-02	8.06E-02	6.92E-02	6.85E-02	6.93E-02
8. Geomembrane (kg)	9.25E-04	9.75E-04	8.80E-04	8.53E-04	8.45E-04	8.54E-04	9.00E-04	8.09E-04	7.81E-04	7.71E-04
9. Limestone (kg)	2.26E-01	2.06E-01	1.71E-01	1.52E-01	1.38E-01	2.08E-01	1.90E-01	1.58E-01	1.39E-01	1.26E-01
10. Water (kg)	3.13E+00	2.86E+00	2.38E+00	2.11E+00	1.92E+00	2.89E+00	2.64E+00	2.19E+00	1.93E+00	1.76E+00
**Operation and maintenance phase**										
11. Rainwater (kg)	1.00E+03	1.00E+03	1.00E+03	1.00E+03	1.00E+03	1.00E+03	1.00E+03	1.00E+03	1.00E+03	1.00E+03
12. Chlorine (kg)	1.00E-03	1.00E-03	1.00E-03	1.00E-03	1.00E-03	1.00E-03	1.00E-03	1.00E-03	1.00E-03	1.00E-03
**End disposal phase**										
13. Coal (kg)	3.90E-01	4.06E-01	3.79E-01	3.73E-01	3.74E-01	3.60E-01	3.75E-01	3.49E-01	3.41E-01	3.41E-01
14. Oil (kg)	5.71E-02	5.95E-02	5.56E-02	5.46E-02	5.48E-02	5.27E-02	5.49E-02	5.11E-02	5.00E-02	5.00E-02
15. Natural gas (m^3)	7.62E-03	7.94E-03	7.42E-03	7.29E-03	7.31E-03	7.03E-03	7.33E-03	6.82E-03	6.67E-03	6.67E-03
16. Electricity (kw·h)	3.11E-01	3.24E-01	3.02E-01	2.97E-01	2.98E-01	2.87E-01	2.99E-01	2.78E-01	2.72E-01	2.72E-01

Table S2. Life cycle assessment list of all scenarios for community-based rainwater harvesting and utilization (continued).

**Floor-area ratio (FAR)**	**1.7**									
**Total runoff coefficient**	**0.09**					**0.20**				
**Reservoir volume (m³)**	**55**	**110**	**165**	**220**	**275**	**55**	**110**	**165**	**220**	**165**
**Construction phase**										
1. PP (kg)	1.69E-01	1.88E-01	1.99E-01	2.16E-01	2.34E-01	1.09E-01	1.18E-01	1.17E-01	1.21E-01	9.64E-02
2. Quartz sand (kg)	2.04E-02	1.95E-02	1.81E-02	1.74E-02	1.70E-02	1.32E-02	1.22E-02	1.06E-02	9.72E-03	6.88E-03
3. PE (kg)	8.62E-04	8.23E-04	7.63E-04	7.35E-04	7.17E-04	5.56E-04	5.17E-04	4.47E-04	4.10E-04	3.27E-04
4. Steel (kg)	6.84E-02	6.54E-02	6.06E-02	5.84E-02	5.70E-02	4.42E-02	4.11E-02	3.55E-02	3.26E-02	3.91E-02
5. PPR (kg)	2.85E-02	2.72E-02	2.53E-02	2.43E-02	2.37E-02	1.84E-02	1.71E-02	1.48E-02	1.36E-02	9.58E-03
6. PVC (kg)	3.31E-04	3.16E-04	2.93E-04	2.82E-04	2.75E-04	2.13E-04	1.98E-04	1.72E-04	1.57E-04	1.89E-04
7. Concrete (kg)	1.68E-01	1.76E-01	1.86E-01	2.01E-01	2.18E-01	1.08E-01	1.11E-01	1.09E-01	1.13E-01	9.00E-02
8. Geomembrane (kg)	1.77E-03	1.91E-03	1.97E-03	2.13E-03	2.24E-03	1.14E-03	1.20E-03	1.16E-03	1.19E-03	1.00E-03
9. Limestone (kg)	2.87E-01	2.75E-01	2.55E-01	2.45E-01	2.39E-01	1.85E-01	1.72E-01	1.49E-01	1.37E-01	1.64E-01
10. Water (kg)	4.00E+00	3.82E+00	3.54E+00	3.41E+00	3.33E+00	2.58E+00	2.40E+00	2.07E+00	1.91E+00	2.28E+00
**Operation and maintenance phase**										
11. Rainwater (kg)	1.00E+03	1.00E+03	1.00E+03	1.00E+03	1.00E+03	1.00E+03	1.00E+03	1.00E+03	1.00E+03	1.00E+03
12. Chlorine (kg)	1.00E-03	1.00E-03	1.00E-03	1.00E-03	1.00E-03	1.00E-03	1.00E-03	1.00E-03	1.00E-03	1.00E-03
**End disposal phase**										
13. Coal (kg)	8.26E-01	9.00E-01	9.38E-01	1.00E+00	1.08E+00	5.33E-01	5.65E-01	5.49E-01	5.60E-01	4.43E-01

14. Oil (kg)	1.21E-01	1.32E-01	1.37E-01	1.47E-01	1.58E-01	7.80E-02	8.28E-02	8.04E-02	8.20E-02	6.49E-02
15. Natural gas (m^3)	1.61E-02	1.76E-02	1.83E-02	1.96E-02	2.10E-02	1.04E-02	1.11E-02	1.07E-02	1.09E-02	8.67E-03
16. Electricity (kw·h)	6.58E-01	7.17E-01	7.47E-01	7.99E-01	8.57E-01	4.25E-01	4.50E-01	4.38E-01	4.46E-01	3.53E-01

Table S2. Life cycle assessment list of all scenarios for community-based rainwater harvesting and utilization (continued).

**Floor-area ratio (FAR)**	**1.7**									
**Total runoff coefficient**	**0.31**					**0.42**				
**Reservoir volume (m^3)**	**55**	**110**	**165**	**220**	**275**	**55**	**110**	**165**	**220**	**165**
**Construction phase**										
1. PP (kg)	9.20E-02	9.91E-02	9.57E-02	9.66E-02	9.94E-02	8.32E-02	8.96E-02	8.56E-02	8.56E-02	8.71E-02
2. Quartz sand (kg)	1.11E-02	1.03E-02	8.68E-03	7.78E-03	7.21E-03	1.01E-02	9.28E-03	7.76E-03	6.90E-03	6.32E-03
3. PE (kg)	4.69E-04	4.34E-04	3.66E-04	3.29E-04	3.04E-04	4.25E-04	3.92E-04	3.28E-04	2.91E-04	2.67E-04
4. Steel (kg)	3.73E-02	3.44E-02	2.91E-02	2.61E-02	2.42E-02	3.38E-02	3.11E-02	2.60E-02	2.31E-02	2.12E-02
5. PPR (kg)	1.55E-02	1.44E-02	1.21E-02	1.09E-02	1.01E-02	1.41E-02	1.30E-02	1.09E-02	9.64E-03	8.83E-03
6. PVC (kg)	1.80E-04	1.66E-04	1.41E-04	1.26E-04	1.17E-04	1.63E-04	1.50E-04	1.26E-04	1.12E-04	1.02E-04
7. Concrete (kg)	9.13E-02	9.29E-02	8.94E-02	9.01E-02	9.26E-02	8.26E-02	8.39E-02	8.00E-02	7.99E-02	8.12E-02
8. Geomembrane (kg)	9.66E-04	1.01E-03	9.48E-04	9.53E-04	9.51E-04	8.75E-04	9.12E-04	8.48E-04	8.45E-04	8.34E-04
9. Limestone (kg)	1.57E-01	1.45E-01	1.22E-01	1.10E-01	1.02E-01	1.42E-01	1.31E-01	1.09E-01	9.72E-02	8.90E-02
10. Water (kg)	2.18E+00	2.01E+00	1.70E+00	1.53E+00	1.41E+00	1.97E+00	1.82E+00	1.52E+00	1.35E+00	1.24E+00

**Operation and maintenance phase**										
11. Rainwater (kg)	1.00E+03	1.00E+03	1.00E+03	1.00E+03	1.00E+03	1.00E+03	1.00E+03	1.00E+03	1.00E+03	1.00E+03
12. Chlorine (kg)	1.00E-03	1.00E-03	1.00E-03	1.00E-03	1.00E-03	1.00E-03	1.00E-03	1.00E-03	1.00E-03	1.00E-03
**End disposal phase**										
13. Coal (kg)	4.50E-01	4.74E-01	4.50E-01	4.48E-01	4.56E-01	4.07E-01	4.28E-01	4.03E-01	3.97E-01	4.00E-01
14. Oil (kg)	6.59E-02	6.95E-02	6.59E-02	6.57E-02	6.68E-02	5.97E-02	6.28E-02	5.90E-02	5.82E-02	5.86E-02
15. Natural gas (m^3)	8.80E-03	9.27E-03	8.80E-03	8.77E-03	8.92E-03	7.96E-03	8.38E-03	7.88E-03	7.77E-03	7.82E-03
16. Electricity (kw·h)	3.58E-01	3.78E-01	3.59E-01	3.57E-01	3.64E-01	3.25E-01	3.42E-01	3.21E-01	3.17E-01	3.19E-01

Table S2. Life cycle assessment list of all scenarios for community-based rainwater harvesting and utilization (continued).

**Floor-area ratio (FAR)**	**2.4**									
**Total runoff coefficient**	**0.09**					**0.20**				
**Reservoir volume (m^3)**	**78**	**156**	**234**	**312**	**390**	**78**	**156**	**234**	**312**	**390**
**Construction phase**										
1. PP (kg)	2.17E-01	2.46E-01	2.67E-01	2.93E-01	3.21E-01	1.29E-01	1.41E-01	1.44E-01	1.52E-01	1.63E-01
2. Quartz sand (kg)	2.70E-02	2.62E-02	2.49E-02	2.43E-02	2.40E-02	1.61E-02	1.50E-02	1.34E-02	1.26E-02	1.21E-02
3. PE (kg)	1.02E-03	9.87E-04	9.38E-04	9.15E-04	9.03E-04	6.05E-04	5.67E-04	5.06E-04	4.75E-04	4.57E-04
4. Steel (kg)	6.22E-02	6.02E-02	5.72E-02	5.58E-02	5.51E-02	3.69E-02	3.46E-02	3.09E-02	2.90E-02	2.79E-02
5. PPR (kg)	3.66E-02	3.54E-02	3.37E-02	3.28E-02	3.24E-02	2.17E-02	2.04E-02	1.82E-02	1.71E-02	1.64E-02
6. PVC (kg)	3.00E-04	2.91E-04	2.76E-04	2.70E-04	2.66E-04	1.78E-04	1.67E-04	1.49E-04	1.40E-04	1.35E-04

7. Concrete (kg)	2.09E-01	2.30E-01	2.49E-01	2.73E-01	2.99E-01	1.24E-01	1.32E-01	1.34E-01	1.42E-01	1.52E-01
8. Geomembrane (kg)	2.15E-03	2.36E-03	2.52E-03	2.78E-03	2.96E-03	1.28E-03	1.35E-03	1.36E-03	1.44E-03	1.50E-03
9. Limestone (kg)	2.61E-01	2.53E-01	2.40E-01	2.34E-01	2.31E-01	1.55E-01	1.45E-01	1.30E-01	1.22E-01	1.17E-01
10. Water (kg)	3.63E+00	3.52E+00	3.35E+00	3.27E+00	3.23E+00	2.16E+00	2.02E+00	1.81E+00	1.70E+00	1.63E+00
**Operation and maintenance phase**										
11. Rainwater (kg)	1.00E+03	1.00E+03	1.00E+03	1.00E+03	1.00E+03	1.00E+03	1.00E+03	1.00E+03	1.00E+03	1.00E+03
12. Chlorine (kg)	1.00E-03	1.00E-03	1.00E-03	1.00E-03	1.00E-03	1.00E-03	1.00E-03	1.00E-03	1.00E-03	1.00E-03
**End disposal phase**										
13. Coal (kg)	1.06E+00	1.17E+00	1.25E+00	1.36E+00	1.47E+00	6.30E-01	6.73E-01	6.76E-01	7.05E-01	7.45E-01
14. Oil (kg)	1.55E-01	1.72E-01	1.83E-01	1.99E-01	2.16E-01	9.22E-02	9.87E-02	9.90E-02	1.03E-01	1.09E-01
15. Natural gas (m^3)	2.07E-02	2.29E-02	2.45E-02	2.65E-02	2.88E-02	1.23E-02	1.32E-02	1.32E-02	1.38E-02	1.46E-02
16. Electricity (kw·h)	8.45E-01	9.34E-01	9.98E-01	1.08E+00	1.17E+00	5.02E-01	5.37E-01	5.39E-01	5.62E-01	5.94E-01

Table S2. Life cycle assessment list of all scenarios for community-based rainwater harvesting and utilization (continued).

**Floor-area ratio (FAR)**	**2.4**									
**Total runoff coefficient**	**0.31**					**0.42**				
**Reservoir volume (m^3)**	**78**	**156**	**234**	**312**	**390**	**78**	**156**	**234**	**312**	**390**
**Construction phase**										
1. PP (kg)	1.63E-01	1.05E-01	1.14E-01	1.12E-01	1.15E-01	1.20E-01	9.37E-02	1.01E-01	9.76E-02	9.86E-02

2. Quartz sand (kg)	1.21E-02	1.31E-02	1.21E-02	1.04E-02	9.50E-03	8.93E-03	1.16E-02	1.07E-02	9.10E-03	8.17E-03
3. PE (kg)	4.57E-04	4.93E-04	4.57E-04	3.92E-04	3.58E-04	3.37E-04	4.39E-04	4.05E-04	3.43E-04	3.08E-04
4. Steel (kg)	2.79E-02	3.01E-02	2.79E-02	2.39E-02	2.19E-02	2.05E-02	2.68E-02	2.47E-02	2.09E-02	1.88E-02
5. PPR (kg)	1.64E-02	1.77E-02	1.64E-02	1.41E-02	1.29E-02	1.21E-02	1.58E-02	1.45E-02	1.23E-02	1.11E-02
6. PVC (kg)	1.35E-04	1.45E-04	1.35E-04	1.16E-04	1.06E-04	9.92E-05	1.29E-04	1.19E-04	1.01E-04	9.08E-05
7. Concrete (kg)	1.52E-01	1.01E-01	1.06E-01	1.04E-01	1.07E-01	1.12E-01	9.00E-02	9.44E-02	9.11E-02	9.20E-02
8. Geomembrane (kg)	1.50E-03	1.04E-03	1.09E-03	1.05E-03	1.09E-03	1.10E-03	9.27E-04	9.68E-04	9.22E-04	9.36E-04
9. Limestone (kg)	1.17E-01	1.26E-01	1.17E-01	1.00E-01	9.18E-02	8.63E-02	1.13E-01	1.04E-01	8.79E-02	7.89E-02
10. Water (kg)	1.63E+00	1.76E+00	1.63E+00	1.40E+00	1.28E+00	1.20E+00	1.57E+00	1.45E+00	1.22E+00	1.10E+00
**Operation and maintenance phase**										
11. Rainwater (kg)	1.00E+03	1.00E+03	1.00E+03	1.00E+03	1.00E+03	1.00E+03	1.00E+03	1.00E+03	1.00E+03	1.00E+03
12. Chlorine (kg)	1.00E-03	1.00E-03	1.00E-03	1.00E-03	1.00E-03	1.00E-03	1.00E-03	1.00E-03	1.00E-03	1.00E-03
**End disposal phase**										
13. Coal (kg)	5.13E-01	5.42E-01	5.23E-01	5.31E-01	5.49E-01	4.57E-01	4.81E-01	4.58E-01	4.57E-01	4.66E-01
14. Oil (kg)	7.51E-02	7.95E-02	7.67E-02	7.78E-02	8.04E-02	6.69E-02	7.05E-02	6.71E-02	6.69E-02	6.83E-02
15. Natural gas (m^3)	1.00E-02	1.06E-02	1.02E-02	1.04E-02	1.07E-02	8.93E-03	9.41E-03	8.95E-03	8.93E-03	9.11E-03
16. Electricity (kw·h)	4.09E-01	4.32E-01	4.17E-01	4.23E-01	4.37E-01	3.64E-01	3.84E-01	3.65E-01	3.64E-01	3.71E-01

Table S2. Life cycle assessment list of all scenarios for community-based rainwater harvesting and utilization (continued).

Floor-area ratio (FAR)	3.1									
Total runoff coefficient	0.09					0.20				
Reservoir volume (m³)	100	200	300	400	500	100	200	300	400	500
**Construction phase**										
1. PP (kg)	2.65E-01	3.02E-01	3.33E-01	3.68E-01	4.06E-01	1.48E-01	1.64E-01	1.71E-01	1.83E-01	1.98E-01
2. Quartz sand (kg)	3.25E-02	3.18E-02	3.06E-02	3.02E-02	2.99E-02	1.82E-02	1.73E-02	1.58E-02	1.50E-02	1.46E-02
3. PE (kg)	1.15E-03	1.13E-03	1.09E-03	1.07E-03	1.06E-03	6.46E-04	6.13E-04	5.59E-04	5.32E-04	5.17E-04
4. Steel (kg)	5.91E-02	5.78E-02	5.56E-02	5.48E-02	5.43E-02	3.31E-02	3.14E-02	2.86E-02	2.73E-02	2.64E-02
5. PPR (kg)	4.49E-02	4.39E-02	4.23E-02	4.16E-02	4.13E-02	2.51E-02	2.38E-02	2.17E-02	2.07E-02	2.01E-02
6. PVC (kg)	2.85E-04	2.79E-04	2.69E-04	2.65E-04	2.62E-04	1.60E-04	1.52E-04	1.38E-04	1.32E-04	1.28E-04
7. Concrete (kg)	2.06E-01	2.34E-01	2.63E-01	2.98E-01	2.86E-01	1.16E-01	1.27E-01	1.35E-01	1.48E-01	1.39E-01
8. Geomembrane (kg)	2.30E-03	2.61E-03	2.87E-03	3.12E-03	3.20E-03	1.29E-03	1.42E-03	1.48E-03	1.55E-03	1.56E-03
9. Limestone (kg)	2.48E-01	2.43E-01	2.34E-01	2.30E-01	2.28E-01	1.39E-01	1.32E-01	1.20E-01	1.14E-01	1.11E-01
10. Water (kg)	3.45E+00	3.38E+00	3.26E+00	3.21E+00	3.18E+00	1.93E+00	1.84E+00	1.68E+00	1.60E+00	1.55E+00
**Operation and maintenance phase**										
11. Rainwater (kg)	1.00E+03	1.00E+03	1.00E+03	1.00E+03	1.00E+03	1.00E+03	1.00E+03	1.00E+03	1.00E+03	1.00E+03
12. Chlorine (kg)	1.00E-03	1.00E-03	1.00E-03	1.00E-03	1.00E-03	1.00E-03	1.00E-03	1.00E-03	1.00E-03	1.00E-03
**End disposal phase**										
13. Coal (kg)	1.29E+00	1.44E+00	1.56E+00	1.70E+00	1.86E+00	7.22E-01	7.82E-01	8.02E-01	8.48E-01	9.04E-01

14. Oil (kg)	1.89E-01	2.11E-01	2.28E-01	2.50E-01	2.72E-01	1.06E-01	1.15E-01	1.17E-01	1.24E-01	1.32E-01
15. Natural gas (m^3)	2.52E-02	2.82E-02	3.05E-02	3.33E-02	3.63E-02	1.41E-02	1.53E-02	1.57E-02	1.66E-02	1.77E-02
16. Electricity (kw·h)	1.03E+00	1.15E+00	1.24E+00	1.36E+00	1.48E+00	5.76E-01	6.23E-01	6.39E-01	6.76E-01	7.20E-01

Table S2. Life cycle assessment list of all scenarios for community-based rainwater harvesting and utilization (continued).

**Floor-area ratio (FAR)**	**3.1**									
**Total runoff coefficient**	**0.31**					**0.42**				
**Reservoir volume (m^3)**	**100**	**200**	**300**	**400**	**500**	**100**	**200**	**300**	**400**	**500**
**Construction phase**										
1. PP (kg)	1.17E-01	1.27E-01	1.28E-01	1.33E-01	1.41E-01	1.02E-01	1.11E-01	1.09E-01	1.11E-01	1.16E-01
2. Quartz sand (kg)	1.44E-02	1.34E-02	1.18E-02	1.09E-02	1.04E-02	1.26E-02	1.17E-02	9.99E-03	9.10E-03	8.53E-03
3. PE (kg)	5.10E-04	4.76E-04	4.17E-04	3.87E-04	3.69E-04	4.46E-04	4.14E-04	3.55E-04	3.23E-04	3.03E-04
4. Steel (kg)	2.61E-02	2.43E-02	2.14E-02	1.98E-02	1.89E-02	2.28E-02	2.12E-02	1.81E-02	1.65E-02	1.55E-02
5. PPR (kg)	1.98E-02	1.85E-02	1.62E-02	1.50E-02	1.43E-02	1.73E-02	1.61E-02	1.38E-02	1.26E-02	1.18E-02
6. PVC (kg)	1.26E-04	1.18E-04	1.03E-04	9.57E-05	9.12E-05	1.10E-04	1.02E-04	8.77E-05	7.99E-05	7.49E-05
7. Concrete (kg)	9.11E-02	9.86E-02	1.01E-01	1.08E-01	9.95E-02	7.97E-02	8.59E-02	8.59E-02	8.98E-02	8.17E-02
8. Geomembrane (kg)	1.02E-03	1.10E-03	1.10E-03	1.13E-03	1.11E-03	8.90E-04	9.57E-04	9.37E-04	9.43E-04	9.12E-04
9. Limestone (kg)	1.10E-01	1.02E-01	8.97E-02	8.32E-02	7.93E-02	9.59E-02	8.91E-02	7.62E-02	6.94E-02	6.51E-02
10. Water (kg)	1.52E+00	1.42E+00	1.25E+00	1.16E+00	1.11E+00	1.33E+00	1.24E+00	1.06E+00	9.69E-01	9.08E-01

**Operation and maintenance phase**										
11. Rainwater (kg)	1.00E+03	1.00E+03	1.00E+03	1.00E+03	1.00E+03	1.00E+03	1.00E+03	1.00E+03	1.00E+03	1.00E+03
12. Chlorine (kg)	1.00E-03	1.00E-03	1.00E-03	1.00E-03	1.00E-03	1.00E-03	1.00E-03	1.00E-03	1.00E-03	1.00E-03
**End disposal phase**										
13. Coal (kg)	5.69E-01	6.07E-01	5.99E-01	6.16E-01	6.45E-01	4.98E-01	5.28E-01	5.08E-01	5.14E-01	5.30E-01
14. Oil (kg)	8.34E-02	8.89E-02	8.77E-02	9.03E-02	9.45E-02	7.30E-02	7.74E-02	7.45E-02	7.53E-02	7.76E-02
15. Natural gas (m^3)	1.11E-02	1.19E-02	1.17E-02	1.21E-02	1.26E-02	9.74E-03	1.03E-02	9.94E-03	1.01E-02	1.04E-02
16. Electricity (kw·h)	4.54E-01	4.84E-01	4.77E-01	4.91E-01	5.14E-01	3.97E-01	4.21E-01	4.05E-01	4.10E-01	4.22E-01

Table S3. Life cycle assessment results of community-based rainwater utilization (ADPF: abiotic depletion potential for fossil fuels, ADP elements: abiotic depletion potential elements, AP: acidification potential, MAETP: marine aquatic ecotoxicity potential, TETP: terrestrial ecotoxicity potential, EP: eutrophication potential, FAETP: freshwater aquatic ecotoxicity potential, GWP: global warming potential, HTP: human toxicity potential, POCP: photochemical ozone creation potential).

**Floor-area ratio (FAR)**		**1**									
**Total runoff coefficient**		**0.09**					**0.20**				
**Reservoir volume (m^3)**		**33**	**66**	**99**	**132**	**165**	**33**	**66**	**99**	**132**	**165**
ADPF	MJ	82.3	91.3	90.5	91.6	95.3	65.2	66.6	62.3	61.8	61.4
ADP elements	kg Sb eq.	9.49E-07	9.73E-07	8.93E-07	8.53E-07	8.38E-07	7.53E-07	7.11E-07	6.20E-07	5.75E-07	5.46E-07
AP	kg SO_2 eq.	0.0454	0.0485	0.0461	0.0458	0.0465	0.0358	0.0352	0.0318	0.0304	0.0298
MAETP	kg DCB eq.	534	566	543	538	550	439	433	401	388	382
TETP	kg DCB eq.	0.0265	0.0276	0.026	0.0247	0.026	0.0217	0.021	0.0191	0.0182	0.0177

EP	kg PO_4^{3-} eq.	0.0014	0.00158	0.00146	0.00145	0.0015	0.000882	0.000858	0.000681	0.000614	0.00058
FAETP	kg DCB eq.	0.0314	0.0336	0.0325	0.0322	0.033	0.0257	0.0255	0.0236	0.0229	0.0226
GWP	kg CO_2 eq.	6.37	6.85	6.59	6.57	6.71	5.09	5.04	4.62	4.46	4.39
HTP	kg DCB eq.	0.949	1	0.956	0.944	0.96	0.783	0.769	0.709	0.684	0.673
POCP	kg Ethene eq.	0.00349	0.00338	0.00322	0.0032	0.00326	0.00251	0.00247	0.00224	0.00215	0.00211
**Total runoff coefficient**				**0.31**					**0.42**		
ADPF	MJ	57.8	58.8	54.6	53.1	52.7	53.8	54.8	50.6	49.1	48.6
ADP elements	kg Sb eq.	6.67E-07	6.27E-07	5.43E-07	4.99E-07	4.71E-07	6.20E-07	5.85E-07	5.05E-07	4.62E-07	4.35E-07
AP	kg SO_2 eq.	0.0316	0.031	0.0278	0.0263	0.0255	0.0293	0.0288	0.0257	0.0243	0.0235
MAETP	kg DCB eq.	398	391	361	347	340	375	371	339	327	319
TETP	kg DCB eq.	0.0196	0.0189	0.0171	0.0162	0.0157	0.0184	0.0179	0.0161	0.0152	0.0147
EP	kg PO_4^{3-} eq.	0.000658	0.000629	0.000462	0.000385	0.000346	0.000536	0.000511	0.000349	0.000274	0.000234
FAETP	kg DCB eq.	0.0232	0.0229	0.0211	0.0203	0.0199	0.0218	0.0216	0.0198	0.0191	0.0187
GWP	kg CO_2 eq.	4.52	4.47	4.07	3.88	3.79	4.22	4.18	3.78	3.6	3.51

HTP	kg DCB eq.	0.71	0.695	0.64	0.613	0.599	0.669	0.66	0.603	0.579	0.564
POCP	kg Ethene eq.	0.00223	0.00218	0.00197	0.00187	0.00182	0.00207	0.00204	0.00183	0.00173	0.00168

Table S3. Life cycle assessment results of community-based rainwater utilization (continued).

**Floor-area ratio (FAR)**		**1.7**									
**Total runoff coefficient**		**0.09**					**0.20**				
**Reservoir volume (m³)**		**55**	**110**	**165**	**220**	**275**	**55**	**110**	**165**	**220**	**275**
ADPF	MJ	113	121	124	130	139	75.3	78.2	75.1	75.7	77.5
ADP elements	kg Sb eq.	1.35E-06	1.32E-06	1.26E-06	1.24E-06	1.24E-06	8.92E-07	8.52E-07	7.63E-07	7.22E-07	6.98E-07
AP	kg SO_2 eq.	0.0635	0.0651	0.0644	0.066	0.0682	0.0418	0.0418	0.0388	0.038	0.038
MAETP	kg DCB eq.	704	721	717	733	757	493	493	466	459	460
TETP	kg DCB eq.	0.0324	0.0327	0.032	0.0323	0.033	0.0226	0.0223	0.0207	0.0202	0.02
EP	kg PO_4^{3-} eq.	0.00238	0.00248	0.00246	0.00255	0.00268	0.00121	0.00122	0.00106	0.00102	0.00103
FAETP	kg DCB eq.	0.0419	0.0432	0.0431	0.0443	0.046	0.029	0.0292	0.0276	0.0273	0.0274
GWP	kg CO_2 eq.	8.73	9.02	9.01	9.28	9.64	5.84	5.88	5.52	5.45	5.48
HTP	kg DCB eq.	1.25	1.27	1.26	1.28	1.32	0.879	0.875	0.823	0.808	0.807

POCP	kg Ethene eq.	0.00429	0.0044	0.00437	0.00448	0.00464	0.00285	0.00285	0.00266	0.00261	0.00261
**Total runoff coefficient**		**0.31**					**0.42**				
ADPF	MJ	64.5	66.6	62.6	61.7	62.2	58.9	60.7	56.7	55.4	55.2
ADP elements	kg Sb eq.	7.62E-07	7.27E-07	6.36E-07	5.91E-07	5.64E-07	6.98E-07	6.63E-07	5.78E-07	5.31E-07	5.01E-07
AP	kg SO_2 eq.	0.0357	0.0355	0.0322	0.0309	0.0304	0.0326	0.0323	0.0291	0.0277	0.0269
MAETP	kg DCB eq.	433	432	401	388	384	402	400	370	357	350
TETP	kg DCB eq.	0.0198	0.0195	0.0179	0.0171	0.0168	0.0184	0.018	0.0165	0.0157	0.0153
EP	kg PO_4^{3-} eq.	0.00088	0.000874	0.000704	0.000635	0.000611	0.00071	0.000701	0.000533	0.000459	0.000422
FAETP	kg DCB eq.	0.0254	0.0255	0.0237	0.0229	0.0227	0.0235	0.0235	0.0217	0.021	0.0206
GWP	kg CO_2 eq.	5.03	5.04	4.64	4.48	4.43	4.61	4.6	4.21	4.04	3.95
HTP	kg DCB eq.	0.774	0.769	0.711	0.686	0.677	0.72	0.712	0.657	0.632	0.617
POCP	kg Ethene eq.	0.00244	0.00243	0.00222	0.00213	0.0021	0.00228	0.00222	0.00201	0.00192	0.00187

Table S3. Life cycle assessment results of community-based rainwater utilization (continued).

**Floor-area ratio (FAR)**		**2.4**	
**Total runoff coefficient**	**0.09**		**0.20**

**Reservoir volume (m³)**		**78**	**156**	**234**	**312**	**390**	**78**	**156**	**234**	**312**	**390**
ADPF	MJ	142	154	162	173	186	87	91.1	90.4	93	97.2
ADP elements	kg Sb eq.	1.70E-06	1.69E-06	1.65E-06	1.64E-06	1.66E-06	1.04E-06	1.00E-06	9.20E-07	8.88E-07	8.74E-07
AP	kg SO_2 eq.	0.08	0.0831	0.0845	0.0876	0.0916	0.0485	0.0489	0.0468	0.0468	0.0477
MAETP	kg DCB eq.	859	894	911	945	985	556	560	542	543	554
TETP	kg DCB eq.	0.038	0.0389	0.0391	0.0401	0.0414	0.0246	0.0244	0.0233	0.0232	0.0234
EP	kg PO_4^{3-} eq.	0.00328	0.00347	0.00356	0.00375	0.00398	0.00158	0.0016	0.0015	0.00151	0.00156
FAETP	kg DCB eq.	0.0515	0.0539	0.0551	0.575	0.0602	0.0329	0.0333	0.0324	0.0326	0.0333
GWP	kg CO_2 eq.	10.9	11.4	11.7	12.2	12.8	6.72	6.82	6.6	6.64	6.8
HTP	kg DCB eq.	1.53	1.58	1.6	1.65	1.71	0.992	0.992	0.956	0.954	0.969
POCP	kg Ethene eq.	0.00531	0.00554	0.00565	0.00588	0.00616	0.00325	0.00329	0.00316	0.00317	0.00324
**Total runoff coefficient**				**0.31**					**0.42**		
ADPF	MJ	71.9	74.6	71.3	71.6	73.2	64.8	66.9	63.1	62.3	63
ADP elements	kg Sb eq.	8.57E-07	8.17E-07	7.27E-07	6.86E-07	6.62E-07	7.72E-07	7.31E-07	6.44E-07	5.99E-07	5.72E-07

AP	kg SO_2 eq.	0.04	0.0399	0.0368	0.0359	0.0358	0.0359	0.0357	0.0325	0.0312	0.0307
MAETP	kg DCB eq.	472	472	444	437	437	433	432	402	390	387
TETP	kg DCB eq.	0.0209	0.0206	0.0191	0.0187	0.0185	0.0191	0.0189	0.0174	0.0167	0.0164
EP	kg PO_4^{3-} eq.	0.00111	0.00111	0.000955	0.000911	0.00091	0.000892	0.000886	0.00072	0.000653	0.000635
FAETP	kg DCB eq.	0.0278	0.0279	0.0263	0.0259	0.026	0.0254	0.0255	0.0237	0.0231	0.0229
GWP	kg CO_2 eq.	5.58	5.6	5.24	5.15	5.17	5.03	5.05	4.66	4.51	4.47
HTP	kg DCB eq.	0.844	0.839	0.786	0.77	0.767	0.774	0.769	0.714	0.689	0.681
POCP	kg Ethene eq.	0.00269	0.00269	0.0025	0.00245	0.00245	0.00243	0.00242	0.00222	0.00214	0.00211

Table S3. Life cycle assessment results of community-based rainwater utilization (continued).

**Floor-area ratio (FAR)**		**3.1**									
**Total runoff coefficient**		**0.09**					**0.20**				
**Reservoir volume (m^3)**		**100**	**200**	**300**	**400**	**500**	**100**	**200**	**300**	**400**	**500**
ADPF	MJ	171	202	200	215	232	98.5	105	106	110	116
ADP elements	kg Sb eq.	2.06E-06	2.16E-06	2.03E-06	2.05E-06	2.07E-06	1.18E-06	1.15E-06	1.08E-06	1.05E-06	1.04E-06
AP	kg SO_2 eq.	0.0967	0.102	0.104	0.109	0.115	0.0553	0.0563	0.055	0.0557	0.0573

MAETP	kg DCB eq.	1020	1080	1100	1150	1210	619	631	620	629	646
TETP	kg DCB eq.	0.0439	0.0456	0.0462	0.0479	0.0498	0.0268	0.0269	0.0262	0.0263	0.0267
EP	kg PO_4^{3-} eq.	0.00418	0.00451	0.00464	0.00493	0.00524	0.00194	0.00201	0.00195	0.002	0.00209
FAETP	kg DCB eq.	0.0611	0.0689	0.0668	0.0703	0.0742	0.0368	0.0377	0.0372	0.0379	0.039
GWP	kg CO_2 eq.	13.1	14.2	14.3	15.1	16	7.59	7.8	7.68	7.84	8.1
HTP	kg DCB eq.	1.81	1.92	1.92	2.01	2.1	1.1	1.12	1.09	1.1	1.13
POCP	kg Ethene eq.	0.00636	0.0068	0.00691	0.00727	0.00765	0.00367	0.00375	0.00368	0.00374	0.00386
**Total runoff coefficient**		**0.31**					**0.42**				
ADPF	MJ	78.9	82.5	80.5	81.7	84.7	69.7	72.5	70	69.2	70.6
ADP elements	kg Sb eq.	9.44E-07	9.06E-07	8.21E-07	7.82E-07	7.61E-07	8.33E-07	7.97E-07	7.98E-07	6.66E-07	6.39E-07
AP	kg SO_2 eq.	0.0442	0.0443	0.0417	0.0412	0.0403	0.0389	0.0389	0.0372	0.0348	0.0346
MAETP	kg DCB eq.	512	515	491	487	493	460	462	482	424	424
TETP	kg DCB eq.	0.0221	0.022	0.0208	0.0204	0.0205	0.0199	0.0198	0.0188	0.0178	0.0177
EP	kg PO_4^{3-} eq.	0.00134	0.00135	0.00122	0.0012	0.00117	0.00105	0.00106	0.00103	0.00085	0.000844
FAETP	kg DCB eq.	0.0302	0.0306	0.0292	0.0291	0.0295	0.0271	0.0273	0.0267	0.0252	0.0252

GWP	kg CO_2 eq.	6.12	6.19	5.89	5.86	5.95	5.42	5.46	5.34	4.99	4.99
HTP	kg DCB eq.	0.914	0.914	0.869	0.856	0.833	0.823	0.821	0.838	0.749	0.745
POCP	kg Ethene eq.	0.00294	0.00297	0.00281	0.00278	0.00271	0.0026	0.00261	0.00263	0.00236	0.00236

Table S4. Normalization results of community-based rainwater utilization.

**Floor-area ratio (FAR)**	**1**									
**Total runoff coefficient**	**0.09**					**0.20**				
**Reservoir volume (m³)**	**33**	**66**	**99**	**132**	**165**	**33**	**66**	**99**	**132**	**165**
ADPF	3.94E-07	4.37E-07	4.33E-07	4.38E-07	4.56E-07	3.12E-07	3.19E-07	2.98E-07	2.96E-07	2.94E-07
ADP elements	4.56E-15	4.68E-15	4.29E-15	4.10E-15	4.03E-15	3.62E-15	3.42E-15	2.98E-15	2.76E-15	2.63E-15
AP	1.36E-13	1.45E-13	1.38E-13	1.37E-13	1.39E-13	1.07E-13	1.05E-13	9.49E-14	9.07E-14	8.9E-14
MAETP	1.04E-12	1.11E-12	1.06E-12	1.05E-12	1.07E-12	8.57E-13	8.46E-13	7.83E-13	7.58E-13	7.46E-13
TETP	9.85E-14	1.03E-13	9.67E-14	9.18E-14	9.67E-14	8.07E-14	7.81E-14	7.10E-14	6.77E-14	6.58E-14
EP	1.06E-14	1.2E-14	1.11E-14	1.1E-14	1.14E-14	6.68E-15	6.5E-15	5.16E-15	4.65E-15	4.39E-15
FAETP	1.54E-14	1.65E-14	1.59E-14	1.58E-14	1.62E-14	1.26E-14	1.25E-14	1.16E-14	1.12E-14	1.11E-14
GWP	1.53E-13	1.65E-13	1.59E-13	1.58E-13	1.62E-13	1.23E-13	1.21E-13	1.11E-13	1.07E-13	1.06E-13
HTP	1.66E-14	1.75E-14	1.67E-14	1.65E-14	1.68E-14	1.37E-14	1.35E-14	1.24E-14	1.2E-14	1.18E-14
POCP	4.02E-14	3.89E-14	3.71E-14	3.68E-14	3.75E-14	2.89E-14	2.84E-14	2.58E-14	2.47E-14	2.43E-14
**Total runoff coefficient**	**0.31**					**0.42**				
ADPF	2.77E-07	2.81E-07	2.61E-07	2.54E-07	2.52E-07	2.57E-07	2.62E-07	2.42E-07	2.35E-07	2.33E-07

ADP elements	3.21E-15	3.01E-15	2.61E-15	2.40E-15	2.26E-15	2.98E-15	2.81E-15	2.43E-15	2.22E-15	2.09E-15
AP	9.43E-14	9.25E-14	8.3E-14	7.85E-14	7.61E-14	8.75E-14	8.6E-14	7.67E-14	7.25E-14	7.01E-14
MAETP	7.77E-13	7.64E-13	7.05E-13	6.78E-13	6.64E-13	7.32E-13	7.25E-13	6.62E-13	6.39E-13	6.23E-13
TETP	7.29E-14	7.03E-14	6.36E-14	6.02E-14	5.84E-14	6.84E-14	6.65E-14	5.99E-14	5.65E-14	5.46E-14
EP	4.98E-15	4.77E-15	3.50E-15	2.92E-15	2.62E-15	4.06E-15	3.87E-15	2.64E-15	2.08E-15	1.77E-15
FAETP	1.14E-14	1.12E-14	1.03E-14	9.95E-15	9.75E-15	1.07E-14	1.06E-14	9.71E-15	9.36E-15	9.17E-15
GWP	1.09E-13	1.08E-13	9.81E-14	9.35E-14	9.13E-14	1.02E-13	1.01E-13	9.11E-14	8.67E-14	8.46E-14
HTP	1.24E-14	1.22E-14	1.12E-14	1.07E-14	1.05E-14	1.17E-14	1.16E-14	1.06E-14	1.01E-14	9.88E-15
POCP	2.57E-14	2.51E-14	2.27E-14	2.15E-14	2.09E-14	2.38E-14	2.35E-14	2.11E-14	1.99E-14	1.93E-14

Table S4. Normalization results of community-based rainwater utilization (continued).

**Floor-area ratio (FAR)**	**1.7**									
**Total runoff coefficient**	**0.09**					**0.20**				
**Reservoir volume (m³)**	**55**	**110**	**165**	**220**	**275**	**55**	**110**	**165**	**220**	**275**
ADPF	5.41E-07	5.79E-07	5.93E-07	6.22E-07	6.65E-07	3.60E-07	3.74E-07	3.59E-07	3.62E-07	3.71E-07
ADP elements	6.49E-15	6.35E-15	6.06E-15	5.96E-15	5.96E-15	4.29E-15	4.10E-15	3.67E-15	3.47E-15	3.36E-15
AP	1.90E-13	1.94E-13	1.92E-13	1.97E-13	2.04E-13	1.25E-13	1.25E-13	1.16E-13	1.13E-13	1.13E-13
MAETP	1.38E-12	1.41E-12	1.40E-12	1.43E-12	1.48E-12	9.63E-13	9.63E-13	9.10E-13	8.96E-13	8.98E-13
TETP	1.20E-13	1.22E-13	1.19E-13	1.20E-13	1.23E-13	8.40E-14	8.29E-14	7.70E-14	7.51E-14	7.43E-14
EP	1.80E-14	1.88E-14	1.86E-14	1.93E-14	2.03E-14	9.17E-15	9.24E-15	8.03E-15	7.73E-15	7.80E-15
FAETP	2.05E-14	2.12E-14	2.11E-14	2.17E-14	2.25E-14	1.42E-14	1.43E-14	1.35E-14	1.34E-14	1.34E-14
GWP	2.10E-13	2.17E-13	2.17E-13	2.24E-13	2.32E-13	1.41E-13	1.42E-13	1.33E-13	1.31E-13	1.32E-13

HTP	2.19E-14	2.22E-14	2.21E-14	2.24E-14	2.31E-14	1.54E-14	1.53E-14	1.44E-14	1.42E-14	1.41E-14
POCP	4.94E-14	5.06E-14	5.03E-14	5.16E-14	5.34E-14	3.28E-14	3.28E-14	3.06E-14	3.00E-14	3.00E-14
**Total runoff coefficient**			**0.31**					**0.42**		
ADPF	3.09E-07	3.19E-07	3.00E-07	2.95E-07	2.98E-07	2.82E-07	2.90E-07	2.71E-07	2.65E-07	2.64E-07
ADP elements	3.66E-15	3.50E-15	3.06E-15	2.84E-15	2.71E-15	3.36E-15	3.19E-15	2.78E-15	2.55E-15	2.41E-15
AP	1.07E-13	1.06E-13	9.61E-14	9.22E-14	9.07E-14	9.73E-14	9.64E-14	8.69E-14	8.27E-14	8.03E-14
MAETP	8.46E-13	8.44E-13	7.83E-13	7.58E-13	7.50E-13	7.85E-13	7.81E-13	7.23E-13	6.97E-13	6.84E-13
TETP	7.36E-14	7.25E-14	6.65E-14	6.36E-14	6.25E-14	6.84E-14	6.69E-14	6.13E-14	5.84E-14	5.69E-14
EP	6.67E-15	6.62E-15	5.33E-15	4.81E-15	4.63E-15	5.38E-15	5.31E-15	4.04E-15	3.48E-15	3.20E-15
FAETP	1.25E-14	1.25E-14	1.16E-14	1.12E-14	1.11E-14	1.15E-14	1.15E-14	1.06E-14	1.03E-14	1.01E-14
GWP	1.21E-13	1.21E-13	1.12E-13	1.08E-13	1.07E-13	1.11E-13	1.11E-13	1.01E-13	9.73E-14	9.52E-14
HTP	1.36E-14	1.35E-14	1.25E-14	1.20E-14	1.19E-14	1.26E-14	1.25E-14	1.15E-14	1.11E-14	1.08E-14
POCP	2.81E-14	2.80E-14	2.55E-14	2.45E-14	2.42E-14	2.62E-14	2.55E-14	2.31E-14	2.21E-14	2.15E-14

Table S4. Normalization results of community-based rainwater utilization (continued).

**Floor-area ratio (FAR)**						**2.4**				
**Total runoff coefficient**			**0.09**					**0.20**		
**Reservoir volume (m³)**	**78**	**156**	**234**	**312**	**390**	**78**	**156**	**234**	**312**	**390**
ADPF	6.79E-07	7.37E-07	7.75E-07	8.28E-07	8.90E-07	4.16E-07	4.36E-07	4.33E-07	4.45E-07	4.65E-07
ADP elements	8.17E-15	8.13E-15	7.93E-15	7.88E-15	7.98E-15	5.00E-15	4.81E-15	4.42E-15	4.27E-15	4.20E-15
AP	2.39E-13	2.48E-13	2.52E-13	2.61E-13	2.73E-13	1.45E-13	1.46E-13	1.40E-13	1.40E-13	1.42E-13
MAETP	1.68E-12	1.75E-12	1.78E-12	1.85E-12	1.92E-12	1.09E-12	1.09E-12	1.06E-12	1.06E-12	1.08E-12

TETP	1.41E-13	1.45E-13	1.45E-13	1.49E-13	1.54E-13	9.14E-14	9.07E-14	8.66E-14	8.62E-14	8.70E-14
EP	2.48E-14	2.63E-14	2.70E-14	2.84E-14	3.02E-14	1.20E-14	1.21E-14	1.14E-14	1.14E-14	1.18E-14
FAETP	2.52E-14	2.64E-14	2.70E-14	2.82E-14	2.95E-14	1.61E-14	1.63E-14	1.59E-14	1.60E-14	1.63E-14
GWP	2.63E-13	2.75E-13	2.82E-13	2.94E-13	3.08E-13	1.62E-13	1.64E-13	1.59E-13	1.60E-13	1.64E-13
HTP	2.68E-14	2.77E-14	2.80E-14	2.89E-14	2.99E-14	1.74E-14	1.74E-14	1.67E-14	1.67E-14	1.70E-14
POCP	6.11E-14	6.38E-14	6.50E-14	6.77E-14	7.09E-14	3.74E-14	3.79E-14	3.64E-14	3.65E-14	3.73E-14
**Total runoff coefficient**			**0.31**					**0.42**		
ADPF	3.44E-07	3.57E-07	3.41E-07	3.43E-07	3.50E-07	3.10E-07	3.20E-07	3.02E-07	2.98E-07	3.01E-07
ADP elements	4.12E-15	3.93E-15	3.50E-15	3.30E-15	3.18E-15	3.71E-15	3.51E-15	3.10E-15	2.88E-15	2.75E-15
AP	1.19E-13	1.19E-13	1.10E-13	1.07E-13	1.07E-13	1.07E-13	1.07E-13	9.70E-14	9.31E-14	9.16E-14
MAETP	9.22E-13	9.22E-13	8.67E-13	8.54E-13	8.54E-13	8.46E-13	8.44E-13	7.85E-13	7.62E-13	7.56E-13
TETP	7.77E-14	7.66E-14	7.10E-14	6.95E-14	6.88E-14	7.10E-14	7.03E-14	6.47E-14	6.21E-14	6.10E-14
EP	8.41E-15	8.41E-15	7.23E-15	6.90E-15	6.89E-15	6.76E-15	6.71E-15	5.45E-15	4.95E-15	4.81E-15
FAETP	1.36E-14	1.37E-14	1.29E-14	1.27E-14	1.27E-14	1.25E-14	1.25E-14	1.16E-14	1.13E-14	1.12E-14
GWP	1.34E-13	1.35E-13	1.26E-13	1.24E-13	1.25E-13	1.21E-13	1.22E-13	1.12E-13	1.09E-13	1.08E-13
HTP	1.48E-14	1.47E-14	1.38E-14	1.35E-14	1.34E-14	1.36E-14	1.35E-14	1.25E-14	1.21E-14	1.19E-14
POCP	3.10E-14	3.10E-14	2.88E-14	2.82E-14	2.82E-14	2.80E-14	2.78E-14	2.55E-14	2.46E-14	2.43E-14

Table S4. Normalization results of community-based rainwater utilization (continued).

**Floor-area ratio (FAR)**					**3.1**					
**Total runoff coefficient**			**0.09**					**0.20**		
**Reservoir volume (m³)**	**100**	**200**	**300**	**400**	**500**	**100**	**200**	**300**	**400**	**500**

ADPF	8.18E-07	9.67E-07	9.57E-07	1.03E-06	1.11E-06	4.71E-07	5.02E-07	5.07E-07	5.26E-07	5.55E-07
ADP elements	9.90E-15	1.04E-14	9.76E-15	9.86E-15	9.95E-15	5.67E-15	5.53E-15	5.19E-15	5.05E-15	5.00E-15
AP	2.89E-13	3.04E-13	3.10E-13	3.25E-13	3.43E-13	1.65E-13	1.68E-13	1.64E-13	1.66E-13	1.71E-13
MAETP	1.99E-12	2.11E-12	2.15E-12	2.25E-12	2.36E-12	1.21E-12	1.23E-12	1.21E-12	1.23E-12	1.26E-12
TETP	1.63E-13	1.70E-13	1.72E-13	1.78E-13	1.85E-13	9.96E-14	1.00E-13	9.74E-14	9.78E-14	9.93E-14
EP	3.17E-14	3.42E-14	3.52E-14	3.73E-14	3.97E-14	1.47E-14	1.52E-14	1.48E-14	1.52E-14	1.58E-14
FAETP	3.00E-14	3.38E-14	3.27E-14	3.45E-14	3.64E-14	1.80E-14	1.85E-14	1.82E-14	1.86E-14	1.91E-14
GWP	3.16E-13	3.42E-13	3.45E-13	3.64E-13	3.86E-13	1.83E-13	1.88E-13	1.85E-13	1.89E-13	1.95E-13
HTP	3.17E-14	3.36E-14	3.36E-14	3.52E-14	3.68E-14	1.93E-14	1.96E-14	1.91E-14	1.93E-14	1.98E-14
POCP	7.32E-14	7.83E-14	7.95E-14	8.37E-14	8.80E-14	4.22E-14	4.32E-14	4.23E-14	4.30E-14	4.44E-14
**Total runoff coefficient**			**0.31**					**0.42**		
ADPF	3.78E-07	3.95E-07	3.85E-07	3.91E-07	4.05E-07	3.33E-07	3.47E-07	3.35E-07	3.31E-07	3.38E-07
ADP elements	4.54E-15	4.36E-15	3.95E-15	3.76E-15	3.66E-15	4.00E-15	3.83E-15	3.84E-15	3.20E-15	3.07E-15
AP	1.32E-13	1.32E-13	1.24E-13	1.23E-13	1.20E-13	1.16E-13	1.16E-13	1.11E-13	1.04E-13	1.03E-13
MAETP	1.00E-12	1.01E-12	9.59E-13	9.51E-13	9.63E-13	8.98E-13	9.02E-13	9.41E-13	8.28E-13	8.28E-13
TETP	8.22E-14	8.18E-14	7.73E-14	7.58E-14	7.62E-14	7.40E-14	7.36E-14	6.99E-14	6.62E-14	6.58E-14
EP	1.02E-14	1.02E-14	9.24E-15	9.09E-15	8.86E-15	7.95E-15	8.03E-15	7.80E-15	6.44E-15	6.39E-15
FAETP	1.48E-14	1.50E-14	1.43E-14	1.43E-14	1.45E-14	1.33E-14	1.34E-14	1.31E-14	1.24E-14	1.24E-14
GWP	1.47E-13	1.49E-13	1.42E-13	1.41E-13	1.43E-13	1.31E-13	1.32E-13	1.29E-13	1.20E-13	1.20E-13
HTP	1.60E-14	1.60E-14	1.52E-14	1.50E-14	1.46E-14	1.44E-14	1.44E-14	1.47E-14	1.31E-14	1.30E-14
POCP	3.38E-14	3.42E-14	3.23E-14	3.20E-14	3.12E-14	2.99E-14	3.00E-14	3.03E-14	2.72E-14	2.72E-14
